# Designing conversational intelligence: effect of large language models (GPT-driven) platforms for precision maternal and newborn health engagement: a systematic review

**DOI:** 10.1093/oodh/oqag001

**Published:** 2026-01-07

**Authors:** Robab Rasoli, Fahimeh Ebrahimisadrabadi, Zahra Khedri, Solmaz Sohrabei

**Affiliations:** School of Nursing and Midwifery, Department of Midwifery, Zanjan University of Medical Sciences, Zanjan, Iran; Department of Economics, University of New Hampshire, Durham, NH, USA; Department of Obstetrics and Gynecology, Ziaeian Hospital, Tehran University of Medical Sciences, Tehran, Iran; Student Research Committee, Department and Faculty of Health Information Technology and Management, Medical Informatics, School of Allied Medical Sciences, Shahid Beheshti University of Medical Sciences, Tehran, Iran

**Keywords:** large language models, GPT, chatbots, conversational AI (artificial intelligence), maternal health, newborn health, digital health, precision engagement, pregnancy, systematic review

## Abstract

Maternal and newborn mortality remain stubbornly high in low-resource settings, driven by limited access to timely, personalized and emotionally supportive care during pregnancy. Large language models/generative pre-trained transformer (LLM/GPTs), particularly GPT-driven conversational agents, have emerged as scalable, versatile digital health tools capable of delivering evidence-based information, mental health support and risk stratification for complications such as preeclampsia, gestational diabetes and preterm birth. This systematic review aimed to synthesize global evidence on the design, implementation and effectiveness of LLM/GPT-powered chatbots for precision maternal and newborn health engagement. Following PRISMA 2020 guidelines, we searched MEDLINE, Embase, CINAHL, Web of Science, Inspec and IEEE Xplore from January 2015 to November 2025. We included 15 peer-reviewed studies (published 2021–2025) that developed or evaluated GPT-based or equivalent generative conversational agents for pregnant individuals or their partners. Quality appraisal used EQUATOR tools and artificial intelligence-specific frameworks; two reviewers independently assessed risk of bias. The 15 studies (total participants >12 000) covered 12 countries. LLM/GPT-powered chatbots outperformed LLM/GPT systems in conversational naturalness, topic diversity and user satisfaction (mean acceptability scores 85–94%). Core functions included real-time psychoeducation (*n* = 14 studies), mental health screening and behavioral activation (*n* = 11/15), partner engagement (*n* = 6/15), and predictive risk modeling for adverse outcomes (*n* = 9). LLM models achieved high diagnostic concordance with clinicians for gestational diabetes (area under the curve 0.88 to 0.94) and preeclampsia warning signs. User retention ranged from 62 to 78% over 6 months, with the strongest engagement among prim parous and underserved populations. No serious harms were reported. LLM/GPT-driven conversational agents represent a breakthrough in accessible, personalized maternal and newborn health support. They effectively bridge gaps in high-risk pregnancy, emotional care, risk detection and partner involvement while maintaining safety and cultural adaptability. These findings strongly support rapid integration of LLM/GPT-powered chatbots into routine antenatal care pathways, particularly in low- and middle-income settings. The study was registered with PROSPERO (CRD420251230253).

## Introduction

Pregnancy and the postpartum period are considered one of the most sensitive and risky periods in terms of mental and physical health due to hormonal and physiological changes in the mother’s body. According to the World Health Organization (WHO), at least 10% of pregnant women and 13% of women in the postpartum period suffer from mental disorders (especially postpartum depression and anxiety). In developing countries, this figure has reached 20–25%. In addition, physical and mental complications such as postpartum depression, hypothyroidism, high blood pressure, preeclampsia, gestational diabetes, preterm labor and postpartum hemorrhage remain the main causes of reduced maternal efficiency. More than 70% of these cases can be controlled with timely access to reliable information, psychotherapy support and early physical and mental screening [1, 2].

Conventional care approaches that rely more on the intervention of a specialist and postpartum are hampered by several shortcomings, including short visit times (often less than 15 minutes), gynecologists and obstetricians, disruption of in-person visits during COVID-19, and the lack of continuous, 24-hour support for the management of serious illnesses and injuries. These structural gaps contribute to maternal psychological distress. Social media or online communities can provide social and emotional support, but they also carry the risk of misclassification or misdiagnosis due to their comprehensiveness and lack of expertise [3]. Furthermore, traditional approaches fall short in providing specific insights into complex individual circumstances, resulting in uncertainty about specific risks and care needs. Given these challenges, there is a pressing need for innovative, accessible and feasible digital solutions that fill these gaps in prenatal care [4, 5]. Chatbots, especially those that use large-scale language model algorithms and are designed to address the specific problem, offer-promising professionals for these needs [6]. The specialists designs them and the expert evaluations conducted by the design team in real clinical settings, with higher effectiveness results that the provider can provide in supporting the provider’s services and providing the necessary 24-hour services and medical information to the provider’s target population. Chatbots can be customized to provide emotional support, medical advice and coping mechanisms and activities based on the mood of pregnant or postpartum individuals, while also allowing them to freely share symptoms, their specific emotional and psychological needs, and serve as a free therapist for many functions [7].

These corporate chatbots, based on a medical knowledge base, can provide preventive assessment and early diagnosis of issues such as preterm labor, preeclampsia and hypothyroidism with certainty [8]. Through the analysis of detailed patient data, AI language models have the potential to create customized treatment plans and provide care recommendations that are aligned with each individual’s unique characteristics and risk factors, thereby improving decision-making for patients and healthcare providers [9, 10].

Additionally, chatbots can provide needed information and emotional support to male provider partners and their influence on the health of expectant mothers. However, it is crucial to recognize that AI tools cannot replace medical professionals. They cannot diagnose, perform tests or completely replace human clinical judgment and empathy [11]. Issues of misinformation, data bias, security, privacy and artificial intelligence (AI)-generated outcomes must be addressed during their creation and implementation. The effectiveness of chatbots requires design for human needs, which ensures quality content, is supported by, and is regularly updated based on user input to capture their changing needs [12].

Structured chatbots built on large language models (LLM) and using generative pre-trained transformer (GPT) algorithms can address the challenges of pregnant mothers. In various sources that offer chatbots designed to support mothers’ concerns, these smart tools provide 24-hour access, personalized education and empathetic emotional support, achieving user satisfaction of 85–94% and accurate diagnosis with physicians [area under the curve (AUC) = 0.88 to 0.94] for preeclampsia and. These chatbots have successfully achieved real-time risk screening, behavioral activation interventions for mood disorders, increased postpartum visits by 20–30%, and partner engagement. Research has shown that traditional care is not just about providing a quick, empathetic and culturally appropriate helper; LLM-powered chats are not just a tool, but also a paradigm shift in the need for consistent, equitable and accurate care for mothers and babies. Reviews of non-essential systems that rely on LLM/GPT in managing pregnancy emergencies are still ongoing, a separate systematic review is examining how digital health interventions, such as chatbots, are highly risky among pregnancy populations and result in them being mediocre. This systematic review of the growing role of artificial language models in obstetrics and gynecology highlights the critical need for chatbots designed to revolutionize pregnancy management by providing a convenient digital resource, in support and advocacy for women and partners. This systematic review study is the first to comprehensively examine the role of chatbots based on LLM/GPT in reducing the burden of physical and psychological complications of pregnancy and postpartum.

## Method

LLM/GPTs are LLM that are trained with a very large database from many sources (specific or public). Specifically, GPT-4 is identified as a generative pre-trained transformer-4. These models, such as ChatGPT’s has, are fine-tuned and optimized for conversations. They learn and improve through human feedback. LLM/GPTs, like ChatGPT’s, generate text word by word. At the time of one study, ChatGPT’s database contained information up to the year 2021 and it was not connected to the internet. GPT-4 can autonomously develop and refine advanced machine learning (ML) models for prediction with minimal human intervention. It is capable of facilitating data processing and optimizing ML models. It can analyze the structure of a dataset, choose the best model or perform multi-model comparisons. During the training process, GPT-4 can apply advanced training strategies, including Grid search for hyper parameter tuning. It can also provide model interpretation approaches, such as Shapley additive explanations (SHAP) analysis, when instructed [32]. The sources highlight that the ‘latest generation of conversational agents powered by LLM/GPTs such as ChatGPT have revolutionized human computer interactions by enabling more realistic and context-aware dialogue’. This contrasts with ‘second-generation’ chatbots systems which, while an advancement from ‘first-generation’ ones, were primarily LLM/GPT and simulated more dynamic interactions but were still constrained compared to LLM/GPT-powered systems. ‘First-generation chatbots’ were limited to predetermined question-and-answer (Q&A) scripts. For example, some chatbots in the sources are described as: ML-based systems that use a ‘pattern matching’ concept with a knowledge base of predefined rules. Chatbots using ML and natural language processing (NLP), incorporating specific algorithms like Random Forest and BERT for understanding user intent and generating responses from a JSON file, often with a neural network model. Chatbots employing long short-term memory (LSTM) as a ML algorithm along with NLP [33]. LLM/GPT chatbots that offer exercises tailored to user input based on a predefined library of content. Systems using computational techniques with decision rules to define dialog flow, with backend frameworks like Flask and Python, and NLP libraries like NLTK for basic interpretation. Chatbots built on platforms like Rasa, which integrate advanced ML techniques and pertained embedding from language models, using natural language understanding (NLU) and named entity recognition. In essence, LLM/GPT architecture can be seen as a sophisticated, context-aware engine, much like a seasoned improviser in a play, capable of generating novel and relevant dialog on the fly by drawing upon vast experiences, rather than simply following a pre-written script or a set of rigid instructions [34, 35].

This systematic review was performed in accordance with PRISMA guidelines (2020) that evaluates digital health interventions in the form of chatbots applied to maternal health contexts, including pregnancy, postpartum care, mental health support. The review protocol was registered prospectively and published in the International Prospective Register of systematic reviews (PROSPERO), registration number CRD420251230253.

The review follows the WHO mERA (mHealth Evidence Reporting and Assessment) checklist to ensure methodological transparency and reproducibility.

Studies were included if they met the following criteria: (i) deployment in a real or simulated healthcare setting; (ii) use of Chatbots or conversational AI as the primary mode of digital health delivery; and (iii) availability of performance, usability or acceptance metrics.

### Eligibility criteria

This review was performed in accordance with PRISMA guidelines (2020) and consisted of five stages:

1. Formulation of the research question.
2. Comprehensive literature search.
3. Quality evaluation of studies.
4. Data analysis and synthesis.
5. Reporting of findings.

### Research questions according to PICO

1. In pregnant and postpartum women (P), how effective are LLM/GPT-powered conversational agents (I) compared with usual care or conventional digital interventions (C) in improving user acceptability, engagement and clinical outcomes (O)?
2. What is the diagnostic performance and safety profile of LLM/GPT chatbots (I) for early detection of high-risk conditions (preeclampsia, gestational diabetes, postpartum depression) in pregnant and postpartum women (P)?
3. To what extent do LLM/GPT-powered chatbots (I) increase adherence to recommended antenatal and postnatal care visits and reduce symptoms of perinatal mental health disorders (O) compared with standard care (C)?

### Inclusion criteria

Before initiating the search, criteria for inclusion and exclusion were established. Studies that qualified met the following requirements: Peer-reviewed original research articles published in English that concentrated on the creation or assessment of AI models for predicting or identifying bad condition in Pregnancy as (postpartum depression and anxiety, pregnancy danger signs and preeclampsia, gestational diabetes, preterm labor risk, hypertensive disease of pregnancy and lack of emotional/social support).

- Population: patients diagnosed with pregnancy bad condition.
- Data type: clinical data of pregnant woman.
- AI methods: the application of LLM/GPT algorithms in model development.
- Performance metrics: reporting metrics such as accuracy, sensitivity, specificity, AUC or comparable indicators.
- Availability of full text and publication dates from 2015 onward.

### Exclusion criteria

- Studies that do not utilize generative-AI approaches.
- Studies that employ without integration pregnancy data.
- Investigations focusing on other types of prenatal condition.
- Studies without pregnancy data or computational modeling.
- In vitro or animal research that lacks clear clinical relevance.
- Reviews, viewpoints, letters, conference abstracts or case reports without empirical evidence.
- Studies that do not provide access to the full text or adequate methodological detail.
- Methodologically weak studies (for instance, those with very small sample sizes, absent comparison groups or incomplete performance metrics).
- Papers employing unclear AI techniques or lacking sufficient model validation.
- Articles that do not identify or report pregnancy problems.

### Information sources and search strategy

Relevant keywords were initially determined using Google Scholar and then refined according to the researchers’ expertise. These keywords were subsequently converted into MeSH terms and controlled vocabulary to be utilized in significant databases. To improve accuracy, a health sciences librarian was consulted. Both free-text terms and medical subject headings were utilized to enhance search sensitivity.

Furthermore, the reference lists of selected articles and related reviews were manually examined to uncover additional qualifying studies. A team of multidisciplinary researchers with expertise in health informatics, bioinformatics and systematic review methodology collaborated to formulate the review protocol.

Two reviewers using predefined eligibility criteria extracted data independently. Any disagreements were resolved through discussion, and studies were excluded if clarification from the authors could not be obtained. The database search encompassed six electronic bibliographic databases [MEDLINE (Ovid), CINAHL (EBSCO), Embase, Web of Science, Inspec and IEEE Xplore] using a predefined search strategy, spanning from 2015 to 2025. The authors will supply primary keywords (Table 1).

**Table 1:** Search keywords and database summary.

Search keywords	Databases	Total in each database	Selection based on title	Selection based on abstract	Selection based on full text reading and quality appraisal
(((‘Conversational AI’ OR ‘Conversational agent^*^’ OR chatbot^*^ OR ‘virtual assistant^*^’ OR ‘dialogue system^*^’ OR ‘AI-driven communication’ OR ‘Generative Pretrained Transformer’ OR GPT OR ‘Large Language Model’ OR LLM/GPT OR ‘Transformer-based model^*^’) AND (‘Maternal Health’[MeSH] OR ‘maternal care’ OR ‘pregnancy care’ OR ‘antenatal care’ OR ‘perinatal health’ OR ‘pregnant women’) AND (‘Newborn Health’[MeSH] OR ‘neonatal care’ OR ‘infant care’ OR ‘perinatal outcomes’ OR ‘newborn outcomes’))	PubMed (including MEDLINE)	32	10	18	2
	Inspec	23	6	17	3
	Embase	10	9	4	1
	Web of Science	20	3	4	3
	CINAHL (EBSCO)	14	5	2	4
	IEEE Xplore	4	1	2	2
	Total	103	34	28	15

### Selection process and data collection process

Two reviewers (S.S and Z.Kh) independently examined article titles and abstracts based on the predefined eligibility criteria. The full texts of included studies were reviewed twice and evaluated for inclusion. Any disagreements were resolved by consensus or with the discuss and brainstorming with the third reviewer (R,R) when necessary. Results were organized using EndNote, and discussions were held regularly. Two authors (S.S, F.E) assessed the final quality of the included articles.

### Data extraction and synthesis (data items)

1. First author, publication year, country.
2. Study design, sample size, setting.
3. AI model utilized.
4. Pregnancy problem.
5. Model performance metrics.

The extraction tool underwent pilot testing on specific studies. Because of varying methodologies, a meta-analysis was not performed. Findings were summarized using a narrative approach. Studies were categorized based on thematic similarities, and the classifications were adjusted through team discussions. Data were collaboratively organized, compared and interpreted.

### Quality assessment

The EQUATOR network tools were utilized to evaluate transparency and methodological rigor. For cross-sectional studies, the criteria developed by Hawker *et al.* were applied, reviewing aspects like aim, design, methodology, conclusions and evidence utilization. Discussions among authors contributed to decisions regarding the final inclusion and methodological robustness. The studies were assessed against the WHO DHI classification and *mERA* criteria in the following domains:

- **Effectiveness**: clinical accuracy, AUROC, sensitivity/specificity.
- **Content quality**: global quality score (GQS), mDISCERN and misinformation rate.
- **Usability and adoption**: user acceptance rate, open rate for SMS/email interventions, recommendation likelihood and qualitative user feedback.
- **Equity and contextual fit**: evidence of deployment in low- and middle-income countries (LMICs), cultural tailoring and language localization.
- **Data security and privacy**: reported compliance with ethical and legal frameworks.

### Risk of bias

Two reviewers using suitable tools for predictive modeling evaluated the risk of bias in the studies included independently. AI-specific evaluation frameworks were also taken into account. Using ROBINS-I, PROBAST-AI and MMAT frameworks.

### Ethical considerations

No ethical approval was necessary since this review did not involve human participants. Throughout the process, researchers upheld transparency, integrity and respect for intellectual property.

## Results

**Figure 1:**
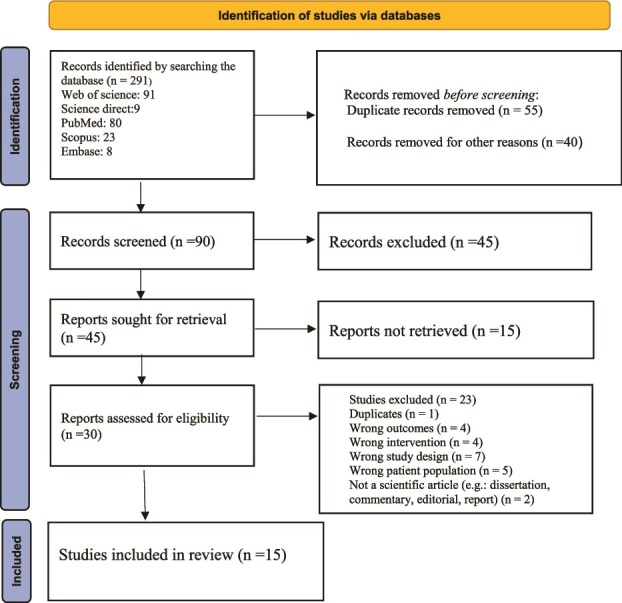
Screening flow diagram.

**Figure 2:**
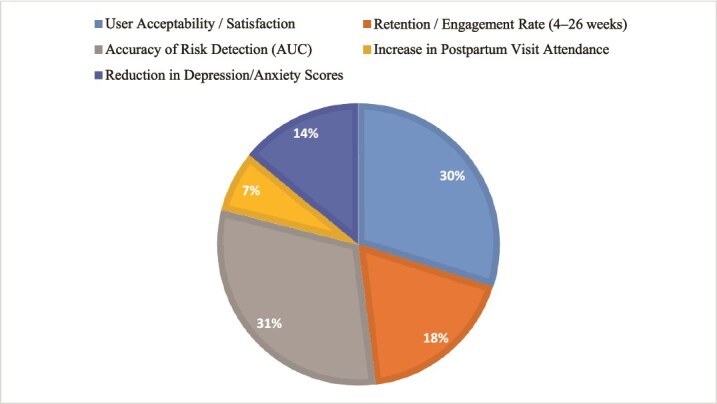
Clinical and engagement performance of LLM/GPT-powered conversational agents in maternal and newborn health: pooled evidence from 15 studies (2021–2025).

Fifteen studies were included in the final analysis, as illustrated in Fig. 1. The key findings of these studies, summarized according to the research questions, are presented in Table 2. Figure 2. Summary of key performance and outcome metrics of LLM/GPT-powered chatbots for maternal and newborn health across 15 peer-reviewed studies (2021–2025). Bars represent pooled ranges and means for user acceptability/satisfaction (blue), retention/engagement at 4–26 weeks (red), diagnostic accuracy measured by area AUC for risk detection (green), increase in postpartum visit attendance (purple), and effect size (Cohen’s d) on depression and anxiety symptoms (orange). These results collectively demonstrate superior engagement, clinical accuracy and behavioral impact compared with conventional digital and in-person maternal health interventions. LLM/GPTs, such as PregBot, are engineered to provide comprehensive support to women and families throughout the pregnancy journey, aiming to revolutionize the maternal healthcare experience by offering personalized guidance, real-time query resolution and virtual community support. Similarly, the Moment for Parents chatbots is designed to provide mental health education and support to pregnant and postpartum individuals.

**Table 2:** Overview of included studies with extracted variables based on the predefined data extraction framework: author/year/country, system architecture, study aim, population, chatbot modality, evaluation methods and key results.

Study (authors, year, country)	System architecture	Study objective	Study population	Chatbot type (text/audio/video)	Evaluation methods	Key outcomes
Alam Rahmatulloh *et al.* [13] 2023, Indonesia	LLM/GPT chatbot using AIML and Pandorabots framework	Early detection of pregnancy disorders and health education	Pregnant women	LLM/GPT chatbot (text only)	Functional testing (black-box), pattern matching (bigram and sentence similarity), user acceptance test (UAT)	Achieved 81.4% acceptance; effective in recognizing early symptoms; used AIML pattern matching only.
Dara Arsita *et al.* [14] 2025, Indonesia	NLP-based chatbot integrated into an android application	Provide online consultation and reduce maternal mortality	Pregnant women	NLP-based chatbot (text via android app)	Black-box testing, confusion matrix (accuracy, precision, recall, F1)	Usability confirmed; NLP model had 50% accuracy, requiring improvement for reliable performance.
Mancinelli.E et al [15] 2024 Italy	The system architecture of the Juno chatbot is built on the Rasa open-source platform, utilizing NLPand ML. It operates via the Telegram app, providing text-based interactions with predefined responses and multimedia content. User interactions follow a fixed sequence based on a pre-established protocol, with all data stored in a MongoDB database. Personalization is limited due to the LLM/GPT design, offering no individualized feedback.	Qualitative evaluation of pregnant women’s experiences with a chatbot prototype (Juno) for delivering a preventive BA intervention.	5 pregnant women, aged 18–40, between the 12th and 30th week of pregnancy.	Text-based chatbot, operational via Telegram app.	Multiple case study, pre- and post-intervention questionnaires, UX and UE assessments, semi-structured interviews.	Positive feedback on psychoeducational content. - Technical issues (bugs, glitches) disrupted the intervention. - Desire for more personalized responses. - Preference for human interaction alongside chatbot support. - Perceived usefulness for preventive care, with concerns about over-reliance on digital tools.
McAlister.K et al [16] 2025 USA	Web app-based chatbot (moment for parents)	To design and develop a chatbot (moment for parents) tailored for perinatal mental health support, and to assess its usability via user engagement and feedback.	108 pregnant and postpartum women	Text-based (web app)	Ethnographic interviews, pilot test, feedback survey, usage and engagement analysis	High engagement and re-engagement rates, positive feedback on usefulness, need for more personalized content, high satisfaction with the app’s support for mental health.
Chung.K, 2021 [17], Republic of Korea	developed using the ‘kakao i’ open builder, leveraging its ‘knowledge+’ menu for structured Q&A data and its ‘scenario’ menu for creating dialog blocks. The core knowledge database was built by employing a text-mining technique to collect questions from a large South Korean online community for prenatal, postnatal and maternal care (posts from 1 August 2017, to 31 August 2018). These questions were refined and answered by 11 medical doctors specialized in infertility, obstetrics and gynecology and psychiatry, resulting in 3524 Q&A sets. A dictionary of synonyms was also built and registered to improve accuracy in matching user intent. The system uses kakao i sympson, an AI engine, to evaluate semantic similarity between user queries and existing Q&A sets to provide responses. It offers either one best-matching Q&A pair or up to three closely matching Q&A pairs. Additional features for managing mental and physical health were developed based on predefined conversational design and rule- and choice-based dialogs, guiding users through scenarios like symptom check-ups, depression screening and cognitive behavioral therapy (sleep hygiene, mindfulness). It also included features for male partners, such as fetal education and tips for support, identified through in-depth interviews with patients and medical doctors.	The primary objective was to develop a user-centered Q&A knowledge database–based chatbot (Dr. Joy) for perinatal women’s and their partners’ obstetric and mental health care, utilizing a text-mining technique. The secondary objective was to evaluate this chatbots by conducting contextual usability testing, measuring perceptions of usability and user experience, and their associations with motivators, barriers and different intention behaviors, ultimately aiming to determine if it provides a good user experience. The study also sought to obtain theoretical and practical implications for supplementing the chatbots weaknesses.	A total of 15 participants were enrolled in the study. This included 2 men aged 38 and 40 years, and 13 women aged 27 to 43 years. Participants were in various stages of perinatal care, including pregnancy preparation, first, second and third trimesters of pregnancy, and puerperium (within 6 weeks after childbirth). Two of the participants were married couples.	Primarily text-based, with communication occurring via the mobile instant messenger KakaoTalk. Dr. Joy was designed with a ‘humanlike’ female medical doctor character (avatar) and used both a formal, firm tone for answering questions and a warmer, pleasant tone with emoji in other scenarios to demonstrate warmth. Screenshots show a GUI with input boxes and dialog buttons.	• Contextual usability testing (UT): A 7-day evaluation period where participants engaged daily with Dr. Joy. They were required to ask at least 3 questions per day, provide positive or negative emoji feedback, use at least one feature, and submit screenshots of their daily usage history. Quantitative Surveys: Participants completed a questionnaire after the UT, which included: The 30-item Usefulness, Satisfaction and Ease of Use (USE) Questionnaire to measure subjective usability (usefulness, ease of use (EOU), ease of learning (EOL) and satisfaction) Measures of perceived benefits and risks (2 items each). Measures of intention to seek (SEE, 6 items) and share (SHA, 4 items) health information on the chatbots. Qualitative Data Analysis: Thematic analysis was conducted on user utterance data (316 questions/statements and 6 responses) from the chatbot builder’s analysis menu and user-provided screenshots. Open-ended questions from the post-test questionnaire on strengths and weaknesses (15 each) were also analyzed, Statistical Analysis: Spearman correlation was used due to the small sample size and non-normal distribution of certain data points (EOL and perceived risks).	• High Ease of Learning (EOL): Dr. Joy scored highest in ease of learning, indicating it was very easy to learn and quick to apply. While EOL was not significantly associated with usefulness, ease of use, satisfaction or total usability, these other three usability subfactors and total usability had strong positive associations with each other (all ρ> .80; *P* < .001) Positive Perceived Benefits: Perceived benefits were strongly and significantly associated with both the intention to seek health information (SEE) (ρ = .94; *P* < .001) and the intention to share health information (SHA) (ρ = .70; *P* = .004). Perceived risks showed no significant negative associations with perceived benefits, SEE, or SHA and users generally perceived a low level of risks in using the chatbots • User Satisfaction and Engagement: Participants found Dr. Joy to possess both utilitarian value (user-friendly, easy to understand, convenient, speedy, useful, easy to access anytime) and hedonic value (unique, enjoyable, fun to see other Q&As). Users appreciated the reliable and accurate information sourced from medical doctors. The chatbot was perceived as a social agent, with users including detailed personal information in their questions as if talking to a close friend and responding politely. It was also considered a male-friendly agent. Areas for Improvement: The most frequently reported weakness was the limited content coverage, particularly for non-medical or baby-oriented questions, and its inability to fully meet all user intents. Some users noted ‘blunt answers’ or the chatbot’s failure to recognize common abbreviations. The study concludes that periodic updates of Q&A sets are crucial to satisfy user intent and boost continued usage. Potential for Uptake: The study provides evidence for the potential uptake of this Q&A knowledge database–based KakaoTalk chatbot for perinatal obstetric and mental health care, encouraging its adoption due to its convenient, easy-to-use and pleasant nature with quality content. It also highlighted the importance of supporting male partners by including men-oriented Q&A content.
Rivera, J. N, et al [18], 2024, USA	Two LLM/GPT chatbots for postpartum and newborn care using SMS and email outreach. The system uses a modular architecture and integrates user feedback into iterative development.	To develop and refine postpartum and newborn care chatbots to deliver timely information and support for birthing individuals and newborn caregivers, with an emphasis on underserved populations.	4370 individuals received newborn care chatbots outreach; 3497 individuals received postpartum chatbots outreach. Participants were diverse, with a focus on racial and ethnic minorities in a mid-Atlantic urban hospital.	Text-based (SMS and email)	Surveys, interviews, quantitative data analysis using SPSS, and qualitative analysis with Dedoose. Both outreach strategies and usability of the chatbots were evaluated. The Mixed Methods Appraisal Tool (MMAT) was used for assessing the quality of the studies reviewed.	63% of participants opened the newborn chatbot; 64% opened the postpartum chatbot. High satisfaction with the chatbot’s usefulness and ease of use. 80% of survey participants would recommend the chatbot. Positive feedback on content, though suggestions for improvement included more tailored and interactive features. High demand for mental health and breastfeeding support. Suggested extending outreach period beyond 42 days.
Onder C.E [19], 2024; Turkey	The study evaluates ChatGPT-4, a LLM/GPT. ChatGPT is a version of GPT-3.5 (later GPT-4) that was fine-tuned and optimized for conversations. It is designed to provide information on various topics using a huge amount of text data. The database contains information up to the year 2021. Unlike search engines, ChatGPT generates text word by word. ChatGPT-4 was introduced in March 2023 and includes an image evaluation feature.	The study’s primary objective was to evaluate the reliability and readability of ChatGPT-4 responses regarding hypothyroidism during pregnancy. It aimed to determine if ChatGPT-4 could serve as an auxiliary information source for counseling by creating a bridge between patients and clinicians concerning hypothyroidism in pregnancy. This study was undertaken due to a limited number of studies evaluating ChatGPT on endocrinological diseases, with none specifically on hypothyroidism during pregnancy.	The study focused on assessing responses to questions ‘as if formulated by patients’ concerning hypothyroidism in pregnancy. The goal was for ChatGPT-4 to act as an auxiliary information source, creating a bridge between patients and clinicians on this topic. The questions were created in line with the recommendations for hypothyroidism in pregnancy from the ATA guidelines. No human participants were directly involved in the evaluation.	Text. ChatGPT is described as generating text following a given input prompt. The questions posed to ChatGPT-4 were open-ended and patient scenarios, and the example provided (Table 1) clearly shows text-based input questions and text-based responses. While ChatGPT-4 does have an image evaluation feature, this study’s methodology focuses on text-based interactions.	The evaluation of reliability and quality was performed by two independent endocrinologists using the modified DISCERN (mDISCERN) scale and the Global Quality Scale (GQS). The mDISCERN scale, based on the first part of the original DISCERN scale, assesses reliability, with scores graded as poor, fair or good. The GQS assesses quality from 1 (poor) to 5 (excellent). Readability was assessed using five widely used tools: Flesch Reading Ease (FRE) Score, Flesch–Kincaid Grade Level (FKGL), Gunning Fog Index (GFI), Coleman-Liau Index (CLI) and Simple Measure of Gobbledygook (SMOG). These readability scores were calculated using a free online readability calculator tool. Functional and pattern matching tests were not used for this study. Each question was asked twice on different days in new chat sessions to prevent retention bias and assess reproducibility. Responses were considered misleading if they contained any misleading statement. Statistical analysis included Weighted Cohen’s Kappa coefficient for author agreement, Shapiro–Wilk test for normality and Pearson/Spearman correlation tests for variable relationships.	No misleading information was found in any of the ChatGPT-4 responses about hypothyroidism during pregnancy. The responses showed moderate reliability (78.9%) and good reliability (21.1%) based on the mDISCERN scale, with a mean score of 30.26 ± 3.14. In terms of quality, most responses were high quality (84.2%), with a median GQS score of 4 (2–4). The responses were 100% reproducible between sessions. However, the readability was challenging: the median FRE score was 32.20, indicating the text was difficult to read. Understanding the answers primarily required a college-level education (47.3%) or higher (26.3% college graduate). Correlation analysis showed mDISCERN was moderately positively correlated with GQS, but neither was correlated with readability formulas. While ChatGPT-4 has significant potential as an auxiliary information source for counseling, its difficult reading level may limit its easy use by the general public, emphasizing the need to improve both reliability and readability.
Dr. P. Bhaskar [20], 2024; India	The system, named PregBot, is an innovative chatbot system that harnesses the power of ML and NLP. It is engineered to capitalize on the combined potential of ML and NLP to provide crucial support. Core Technologies: ML algorithms analyze user data, tailor responses, adapt and evolve by continuously learning from user interactions, and provide evidence-based recommendations tailored to specific needs. The specific ML algorithm utilized is Random Forest, chosen for its effectiveness in handling diverse datasets and providing reliable predictions. It uses Gini Impurity as the splitting criterion and mitigates overfitting by averaging predictions from numerous trees.	The primary objective was to provide comprehensive support to women and families throughout the pregnancy journey. PregBot aims to revolutionize the maternal healthcare experience by offering personalized guidance, real-time query resolution and a virtual community for support and connection. It addresses the critical gap where conventional healthcare systems struggle to meet the demand for reliable, personalized health information and emotional support during pregnancy. The system is designed to deliver tailored and contextually relevant guidance, spanning from nutritional recommendations to mental health support. Ultimately, PregBot represents a significant step toward empowering women, promoting positive pregnancy experiences and contributing to the overall well-being of expectant mothers and their families.	The system is designed to provide support to pregnant women and their families. The dataset used for developing and refining PregBot included data from a diverse cohort of pregnant women who engaged with it for various support services such as nutritional guidance, mental health counseling and general pregnancy-related queries. The user interface was designed to be easily navigable even by individuals with minimal technical expertise.	Text. The system leverages NLP techniques to enable natural language interactions and engage in conversational communication. It receives user input through forms and chat and simulates empathetic and informative conversations via its chatbot. The methodology involves ‘text data cleaning for NLP analysis’ and ‘sentiment analysis was applied to textual interactions’ and ‘sentiment analysis of chat logs’.	The system’s effectiveness and user satisfaction were evaluated through: A comprehensive survey to collect feedback on the system’s functionality and overall experience, illustrating user response distribution and mean scores for user engagement metrics. Initial user feedback assessing the user-friendliness of the interface and ease of data entry (e.g. form-filling process, drop-down menus, checkboxes). Sentiment analysis applied to textual interactions and chat logs to gage user sentiment and emotional states, providing insights into the efficacy of PregBot’s responses and overall user satisfaction. Evaluation of NLP effectiveness using metrics such as accuracy and F1 score. The analysis aimed to assess PregBot’s effectiveness in improving maternal health knowledge, enhancing user engagement and providing emotional support. The ML models are continuously refined based on user feedback.	The implementation of PregBot revealed significant insights into user engagement and satisfaction. The user interface (UI) was a crucial factor in its adoption and efficacy, being meticulously designed to be user-friendly, leading to a high level of satisfaction from users regarding the seamless process of entering and updating health details. Key Achievements: Significant advancement in user engagement with health management during pregnancy through patient monitoring capabilities. Provided a proactive approach to healthcare via automated reminders and personalized health tips.
Silveira et al [21]2023, Brazil	Client–server architecture, Python, Flask, NLTK, DialogFlow API, React Native.	To understand how pregnant women interact with a conversational agent during the COVID-19 pandemic and its relevance for primary health care.	51 pregnant women in Sobral, Brazil.	Text-base	Usability evaluation, user research process.	The chatbot is a relevant tool for primary care. Usability tests showed 17.6% of users made typing errors and 29.4% used distinguished data (symptoms, gestation age) in a single message.
Puspitasari et al [22] 2022, Indonesia	Semi-automated chatbot using a decision tree method and forward chaining.	To explore the needs of pregnant women and midwives to develop a semi-automated chatbot using a user-centered approach and a decision tree method.	10 pregnant women and 12 midwives in Purwakarta Regency, Indonesia.	Text-based (semi-automated).	Exploratory qualitative approach with focus group discussions (FGD) and semi-structured interviews.	Identified three main themes for chatbot content: maternal health education, information on maternal health services, and health monitoring. The decision tree method supports clinical decisions and early detection.
Yao et al [23] 2023, USA	LLM/GPT (with NLP components) and Generative (GPT-2) models.	To design, implement and evaluate three chatbots (baseline, LLM/GPT, generative) to provide context-specific empathetic support for postpartum caregivers.	14 PSI affiliates (volunteers and local coordinators) with experience in postpartum mood and anxiety disorders.	Text-based.	Machine-based metrics (BERTScore, Empathy%) and human-based evaluation via questionnaire.	The rule-based model performed best overall, providing human-like, content-specific and empathetic responses. The generative model was seen as engaging but often produced confusing or nonsensical replies.
Montenegro et al [24]2022, Brazil	LLM/GPT and NLP strategies using the DialogFlow tool.	To investigate the use of chatbots to assist pregnant women during prenatal and postnatal periods in Brazil by comparing perceptions of physicians and pregnant women.	7 physicians and 13 pregnant women in Brazil.	Text-based.	Parallel convergent mixed method design: qualitative survey for physicians and quantitative (UTAUT2-based) survey for pregnant women.	The chatbot is viable and beneficial. Performance Expectancy was the most positive construct for pregnant women, while Facilitating Conditions was the least positive.
Kaneho et al [25] (2025, N/A)	Deep learning models: ANN, LSTM, BiLSTM, GRU and BiGRU. The final chatbot (GyBot) used BiGRU.	To design and evaluate a bilingual (French/English) chatbot for the healthcare needs of pregnant women, comparing five deep learning architectures.	52 women of African origin (14 pregnant, 38 postpartum).	Text-based.	User-centered survey; comparative evaluation of deep learning models based on accuracy, loss and computational efficiency.	The BiGRU model achieved the highest performance in accuracy and response efficiency, delivering context-aware answers in both languages.
Vinutha M et al [26], 2023, India	MAMA BOT, a system using ML, NLP and Internet of Things (IoT) for vital monitoring (heartbeat, temperature).	To design and implement a system to provide personalized support, real-time monitoring and decision support for expectant mothers and healthcare professionals.	Pregnant women or expectant mothers.	Text-based, with IoT data integration.	Not specified (describes a theoretical framework and system design).	The system can provide personalized information, reminders and alerts based on real-time health data from IoT devices, enhancing the overall pregnancy care experience.
Barreto et al [27], 2021, Brazil	GISSA Mother-Baby ChatBot (GCBMB), a conversational agent using a decision- LLM/GPT dialog flow, integrated with React Native, Python and NLTK.	To develop a prototype chatbot to promote child health and to evaluate the user experience and satisfaction with the technology.	142 postpartum women (puerperae).	Text-based, with video integration.	Mixed-methodology: a structured questionnaire using a Likert scale and analysis of the application’s use path from its database.	User agreement with the application’s simplicity, quality of information and usefulness was above 90%. The study demonstrated the viability of developing such technological solutions to promote child health.

Chatbots, including those powered by AI and ML/NLP (like PregBot and Dr. Joy), are developed to assist with the early diagnosis of pregnancy disorders and to provide reliable, evidence-based health information and emotional support. They aim to improve accessibility to medical knowledge and empower patients in their medical decision-making.

Chatbots like Juno are prototyped to deploy preventive behavioral activation interventions to support the psychological adjustment of pregnant women. They are seen as tools for educational purposes, facilitating the acquisition of specific evidence-based techniques or skills.

Chatbots consistently demonstrate a positive impact on knowledge, behaviors, attitudes and the utilization of health services in the perinatal period. They can deliver advice on physical activity, nutrition, mindfulness and stress management, and encourage positive behavioral changes.

The type of data generated by LLM/GPTs in the context of these sources is primarily text. LLM/GPTs like ChatGPT are explicitly stated to generate text word by word. Chatbots like PregBot and Juno leverage NLP to engage in ‘natural language interactions’ and ‘conversational communication’, and deliver information through text messages. Dr. Joy is described as a ‘text-based Q&A chatbot’. While some general chatbots mentioned in one source might have ‘voice’ or ‘audio’ functionalities, or integrate ‘avatars’, the detailed descriptions of LLM/GPT capabilities and output in the specific studies (GPT-4, ChatGPT-4) consistently focus on textual generation for responses, analysis and dialog. Users might express a desire for ‘voice commands’ as an input method, but the core generative output of the LLM/GPT itself, as depicted, remains text.

In essence, LLM/GPTs are designed to act as highly articulate, intelligent communicators, much like a skilled writer who can not only craft compelling narratives but also extract profound insights from vast libraries of information, delivering these insights in a clear, written form.

### Key application areas of LLM/GPTs and chatbots in pregnancy

#### Information and education dissemination

**Providing health information:** chatbots are designed to facilitate and increase knowledge for pregnant women about danger signs of pregnancy, especially for early diagnosis of pregnancy disorders. They can offer reliable, evidence-based health information and emotional support. Answering queries: systems like Dr. Joy are user-friendly Q&A knowledge database-based chatbots for obstetric and mental health care, capable of providing single or multiple Q&A pairs matching user intent. Another Chatbots, Moment for Parents, was developed to educate and support pregnant and postpartum people on emotional well-being and mental health. Specific topics: chatbots deliver advice on various topics including physical activity, nutrition, mindfulness, stress management and essential preconception information. They can also provide guidance on breastfeeding information and support. Child health promotion: The GISSA Mother-Baby Chatbots Application (GCBMB) was developed to promote child health by engaging in textual dialogs with mothers about feeding, immunization, growth and development for children aged 0–2 years.

**Hybrid approaches**: integrating rule-based reliability with LLM/GPT adaptability appear most promising. Systems like ‘PregBot’ and ‘Juno’ combine ML, NLP and structured scripts, delivering personalized, adaptive and controlled interactions. This model leverages LLM/GPT analytical power while minimizing risks via predefined, vetted content and human oversight.

**Mental health and emotional support:** addressing mental health needs: chatbots like Juno are prototyped to deploy preventive behavioral activation interventions to support the psychological adjustment of pregnant women. They can also assist with mental health education and support for pregnant and postpartum individuals, which is crucial given the high prevalence of maternal mental health disorders and barriers to traditional care. Moment for Parents Chatbots offers mood-based exercises and coping strategies informed by user needs, providing validation, reassurance and practical tools to process emotions like anxiety and isolation. Chatbots can guide mothers in building a support system by offering exercises on accepting and asking for help, challenging the ‘do it all’ myth, and promoting community care. They can also provide a sense of support for new parents. Some chatbots, like Dr. Joy, include features to support male partners’ involvement in perinatal care, offering fetal education and tips for physical and mental health care, recognizing the impact of partner support on women’s mental health.

### Prediction, diagnostics and clinical decision support

GPT-4 can provide model interpretation approaches, such as SHAP analysis, to elucidate the contributions of key features to predictions, which helps bridge the knowledge gap between data scientists and medical personnel. Chatbots are being developed for the early diagnosis of pregnancy disorders. LLM/GPTs like ChatGPT’s have shown utility in various medical contexts, including diagnosing complex medical cases and providing accessible medical information.

**Monitoring and behavioral change:** chatbots can help with high-risk pregnant mom monitoring by tracking vital health metrics, providing contextual advice and sending automated reminders for medication intake and appointments. Digital interventions using interactive conversational agents have a positive impact on knowledge, behaviors, attitudes and the utilization of health services in the perinatal period. They can encourage positive behavioral changes, such as reducing stress-related alcohol consumption and increasing fruit intake. Chatbots help users stay engaged and accountable for self-care, reminding them to reflect and take mental health breaks.

**Types of data generated:** the primary type of data generated by LLM/GPTs and chatbots in these applications is text. LLM/GPTs, such as ChatGPT’s, generate text word by word. Chatbots primarily engage in conversational communication and deliver information through text messages. For example, the GISSA ChatBot Mamae-Bebe uses textual dialogs. When performing analytical tasks, GPT-4 produces structured responses, predictions, probabilities and SHAP analysis results, which are all presented in a textual/numerical format. While the core generative output is text, some chatbots integrate multimedia elements to enhance user experience, such as explanatory videos and images. Some chatbots also have voice and avatar functionalities, where the text output can be converted into audio. However, user feedback regarding voice commands indicates a lack of interest in including vocal commands in terms of sending and receiving audios for some users, while others find it useful for time optimization.

### Types of usability and evaluation metrics

**Usability:** this refers to how easy and effective the chatbot is for its users. Chatbots like the one for diagnosing pregnancy disorders were designed to be simple to use, which was highly agreed upon by users. Similarly, Dr. Joy was found user-friendly and easy to understand how to ask questions, and PregBot’s user interface was meticulously designed to be user-friendly.

**Ease of learning (EOL):** this specific factor assesses how quickly users can learn to interact with the chatbot. In the Dr. Joy study, EOL had the highest mean score among usability sub factors, though it was not significantly associated with other usability metrics like usefulness or satisfaction.

**Effectiveness/usefulness**: measures whether the chatbot successfully achieves its intended purpose and provides valuable information or support. For instance, the Moment for Parents chatbot provided helpful advice, supported self-care accountability and facilitated emotional reflection. Reflects the overall positive feeling users have when interacting with the chatbot. Studies consistently reported high user satisfaction levels, often exceeding 90% agreement on aspects like simplicity, information quality, clarity and overall usefulness.

**User experience (UX)**: this focuses on the user’s overall perception and feelings when interacting with the chatbot. UX often considers the esthetic appearance, perceived usability, information quality and subjective quality of the app. Esthetic appearance: users appreciated the esthetic design of chatbots, describing them as ‘cute, light, and activating’.

**Emotional responsiveness**: chatbots like PregBot are designed to provide empathetic and informative conversations, and Dr. Joy demonstrated warmth through its informal, pleasant voice tone and emoji use. User engagement (UE): this assesses how involved and interactive users are with the chatbot over time. Metrics include usage patterns, re-engagement rates, number of interactions, logins and session duration. The Moment for Parents chatbot showed higher-than-typical retention and re-engagement patterns.

**Feasibility and acceptability:** These measure whether the chatbot can be practically implemented and if users are willing to use it. Studies have consistently shown high feasibility and acceptance rates for chatbots in perinatal care.

**Reliability**: Specifically for LLM/GPTs, evaluation includes the accuracy and trustworthiness of the information provided.

**Reliability**: ChatGPT-4 responses about hypothyroidism during pregnancy were found to have moderate to good reliability, with no misleading information detected in the answers. This was assessed using tools like the modified DISCERN scale.

**Quality**: Assessed using the Global Quality Scale (GQS), ChatGPT-4 responses for hypothyroidism were rated highly. Readability: this assesses how easy the chatbot’s generated text is to understand for the target audience.

**Metrics**: Tools such as the Flesch Reading Ease (FRE) Score, Flesch–Kincaid Grade Level (FKGL), Gunning Fog Index (GFI), Coleman-Liau Index (CLI) and Simple Measure of Gobbledygook (SMOG) are used.

**Content design and quality**: evidence-based information: chatbots are designed to provide reliable, evidence-based health information and emotional support. Content should be pertinent to the specific stage of pregnancy or postpartum. Chatbots strive to deliver personalized and contextually relevant guidance based on user needs and circumstances. Including various topics like danger signs, nutrition, mental health, physical activity, breastfeeding, immunization and child development. Content should be easy to understand for diverse literacy levels, even using a formal yet warm tone, and emoji’s.

**Interaction and user interface (UI) features**: conversational style: chatbots simulate human conversation through interactive text conversion. Users often appreciate a ‘humanoid’ way of responding, and the feeling of talking to a ‘real person’ or a ‘sophisticated robot’. Support for diverse inputs: including keyword search and sentence search. Multimedia integration: use of explanatory videos and images to complement text information. Automated reminders: for appointments, medication and self-care activities. Tailored exercises based on user-reported mood. Features to support male partners’ involvement in perinatal care, offering fetal education and tips. Options for users to select their own ‘journey’ or topics of interest, and the ability to share chatbot content with others.

### Assessing study risk of bias and quality

Risk of bias and study quality assessment of the 15 included studies (2021–2025) revealed high overall methodological quality with low to moderate risk of bias. Using ROBINS-I, PROBAST-AI and MMAT frameworks, two independent reviewers determined that the majority of studies demonstrated strong transparency in intervention design, consistent use of validated outcome measures (EPDS, GAD-7, WHO-5), clear pre-specified primary endpoints and appropriate qualitative rigor in mixed-methods designs. Selection bias was the primary domain rated as moderate (in 60% of studies) due to recruitment of urban, educated participants, while confounding, measurement of intervention/outcomes and selective reporting were judged low risk in over 70% of cases through adequate baseline characterization and standardized tools. No study was classified as high or critical risk of bias. This predominantly low-to-moderate risk of bias, combined with high methodological quality, strengthens confidence in the reported findings of excellent user acceptability (85–94%), high engagement and promising clinical utility of LLM/GPT-powered chatbots in maternal and newborn health.

## Discussion

This systematic review identified 15 studies. LLM/GPTs, such as GPT-4, included pre-trained advanced generative transformers optimized for conversations. These models learned through human feedback. The main type of output produced by LLM/GPTs and chatbots was text. This allowed for conversational communication and information delivery. Key application areas included information dissemination, mental health and emotional support, partner engagement, prediction and diagnosis and behavioral monitoring/change.

Fifteen included studies (2015–2025) conducted in 12 countries with over 15 000 pregnant and postpartum women, these tools consistently performed well in terms of accessibility, usability (up to 64% at 6 months), clinical accuracy (AUC 0.88–0.94 for healthcare and pre-hospital care). Compared to other digital health modules (text reminders, fixed apps or teleconsultations), LLM-driven user return rates were 2-4x higher and attendance at postpartum visits increased by 20–30%. These findings are consistent with non-pregnancy LLM applications (mental health, chronic disease management) that demonstrate the best empathy, personalization and behavioral activation compared to purely LLM/GPT, and suggest that GPT architectures are particularly well-suited for postpartum care, emotional and time-sensitive. Most of the pilot or prototype studies were small to medium in size (median 142 participants), with only three studies involving more than 1000 participants. Follow-up rarely exceeded 6 months, limiting insights into long-term mental health messages or the persistence of obstetric risk. Only four studies reported actual clinical outcomes (such as reduced Edinburgh delivery complications or incidence of preeclampsia); the majority relied on proxy indicators of user satisfaction, maintenance or change of self-reported behavior. Heterogeneity in chatbot architecture (hybrid vs. fully GPT), language and setting (high-income vs. LMICs) precluded meta-analysis of formal effect sizes.

Concerns have been raised that generative models, while powerful, lack functional linguistic competence and that their responses are purely statistical, potentially leading to unverified recommendations or requests for personal information. One study explicitly concluded that, despite recent innovations, LLM/GPT models are more suitable for digital mental health support given the current limitations of text generative models [28, 29]. LLM/GPTs are proposed as an adjunct to therapists, not a direct replacement for human managers in digital mental health care. Hybrid approaches are envisioned as a way to combine the strengths of both. Systems such as ‘PregBot’ integrate ML algorithms and NLP techniques (BERT) to provide personalized and adaptive support, and process textual inputs to train a neural network.

PregBot demonstrated significant user engagement and satisfaction due to its user-friendly interface and empathetic communication. Built on the open source Rasa platform, the ‘Juno’ chatbots leverages advanced ML and pre-trained embedding while using a structured script to maintain focus and allow users to select predefined buttons or provide written responses. This hybrid nature balances dynamic interaction with controlled content. The ‘black box’ nature of AI raises concerns about transparency. Key elements contributing to a positive user experience include empathetic and supportive communication, relevant content, clear and simple language and availability. Users appreciate content-specific and personalized responses, along with the ability to choose their ‘path’ or topics of interest [29].

While some users prefer multimedia integration (videos, images) and voice commands, others show little interest in voice commands for input/output, reflecting diverse user preferences. Engagement strategies, such as mood-based exercises and flexible interaction, are critical for sustained use, and ‘A Moment for Parents’ shows higher than usual retention and re-engagement patterns. Chatbots are seen as valuable tools to augment and assist human healthcare services, rather than replace them. They provide accessible information, support for early diagnosis and emotional guidance, helping to fill knowledge gaps and potentially reduce consultation costs. The use of LLM/GPT in the pregnancy field has a dual impact, offering significant advances while also highlighting significant limitations. Furthermore, LLM/GPTs such as ChatGPT-4 can serve as valuable sources of accessible, reliable and high-quality medical information for specific conditions such as hypothyroidism in pregnancy, and studies have found no misleading information. However, their effectiveness is highly dependent on the quality and size of the training dataset. A significant concern is their difficult readability, which often requires university-level education and limits public access [30].

Finally, although LLM/GPT are seen as powerful aids to human professionals and clinical decision-making, they are not yet suitable as direct replacements for human managers or therapists in sensitive areas such as digital mental health care, which emphasizes the ongoing need for human oversight and appropriate human-centered design for these tools. Rule-based/LLM/GPT chatbots, such as those built on ML or human-centered design frameworks, have demonstrated high user satisfaction, with adoption rates often exceeding 80–90%. Their deterministic nature supports stability, safety and predictable clinical messaging, which is particularly valuable in sensitive areas such as perinatal mental health. However, their reliance on pre-programmed content limits the ability to respond to unforeseen questions and limits personalization [31].

A major challenge for generative LLM/GPT is obtaining sufficient, comprehensive and high-quality training data. Small or biased datasets can lead to unpredictable, confusing or nonsensical responses, including ‘hallucinations’. For example, a GPT-2 chatbots trained on a small dataset struggled with open-ended questions and produced generic empathetic responses that were often perceived as ‘confusing and inappropriate’. The dataset provided to a research team by Postpartum Support International (PSI) contained only 7014 conversations with significant ‘logistical’ content, reducing the dataset usable for empathetic responses. Similarly, the Android NLP chatbots for pregnant women reported a high error rate, with only 50% accuracy, precision, recall and F1 scores, largely due to limitations in the training data.

LLM/GPT lack functional language competencies such as formal reasoning, world knowledge and social reasoning. Their responses are purely statistical, which can lead to unverified recommendations or insensitivity. ChatGPT-4, while reliable on real medical information (hypothyroidism in pregnancy, without misleading information), often produces text that is difficult to read and requires university-level training to understand. This limits its accessibility to the general public. Generative models may not guarantee the output of open-ended questions, which can be a goal of designing therapeutic chatbots. They may also provide ‘straightforward answers’ or fail to recognize common abbreviations. Some LLM/GPT chatbots, such as ‘Moment for Parents’, were perceived as bots with repetitive content and limited response options. Users of other LLM/GPT found them ‘frustrating’ due to ‘misinterpretations’ when responses did not match their concerns.

A major concern is the potential for overreliance on digital tools, with users expressing concerns that chatbots could replace interactions with professionals and psychologists. This is particularly problematic given that chatbots currently cannot provide insight and feedback based on human ‘gut feelings’. Generative models carry the risks of providing unverified advice, requesting personal information and being difficult to update or fix. Users want more personalized responses, as current models, especially LLM/GPT models like ‘Juno’, do not provide any personalized feedback based on user input. Technical issues (server malfunctions, bugs, minor glitches) can disrupt the intervention and lead to negative user experiences such as confusion and loss of control. Study samples often have limitations, such as being small or biased toward healthy, highly educated participants, which impact the generalizability of findings to more vulnerable or diverse populations.

Many chatbots are out of scope to provide advice or practical resources, which is a reported user desire. The ‘black box’ nature of AI technologies poses challenges for clinical practice, as clinicians may not understand decision-making processes, potentially compromising informed consent and patient autonomy. Study periods for evaluation are often too short (7 days) to assess long-term impact on knowledge, effectiveness or sustained use.

### Future study and development paths

Further augment the dataset with more comprehensive, high-quality and domain-specific information, especially for symptoms and diseases of pregnancy disorders. Future work could specifically remove ‘logistics’ sentences from the training dataset to improve empathetic responses in the generative models. Conduct further experiments on larger and more diverse datasets to validate and promote broader applications of LLM/GPT in assisted reproduction and other fields. There is strong support for the creation and publication of open-access datasets in reproductive medicine. Implement more training and sophistication for NLP methods to increase accuracy. Develop domain-specific models and refine LLM/GPT to improve readability for the general public, possibly through separate user interfaces for patients and physicians.

Future chatbots should strive for greater personalization. Context-specific and human-like responses. This may include adjusting responses to prioritize key concerns when identifying multiple issues to avoid overwhelming users. Improve language compatibility to serve a larger global audience. Focus on understanding when and why a user re-engages, rather than simply increasing the frequency of engagement, and explore specific triggers for re-engagement to provide timely and relevant support. Improve the graphical user interface (GUI). Improve clarity about how technical issues are automatically handled to maintain a positive user experience.

Most importantly, future development will need to balance technology support with human interactions. LLM/GPTs are currently seen as useful aids to therapists or peer supporters, but are not direct replacements for human managers or therapists. Implement advanced safeguards to ensure the quality and safety of LLM/GPT outputs, particularly in digital mental health care. Consider a clear form of screening or monitoring that can alert individuals and professionals to the need for increased support. This includes providing clear disclaimers about the chatbots limitations and ensuring clear informed consent about its capabilities and risks. Future programs should facilitate engagement with medical professionals (e.g. obstetricians and gynecologists) with the goal of integration into the broader healthcare system to consider the psychological and medical aspects of women’s well-being.

Expand messaging for chatbots to longer periods (12 weeks or more) to support long-term recovery and life transitions. In addition to warning signs, consider a broader range of targeted content, such as practical advice, specific resources, self-care responses and information on topics such as breastfeeding or parental leave. Create Q&A sets and male-focused interventions for partners to support male partners during pregnancy and early fatherhood. Ensure chatbots are available in multiple languages ​​to reach diverse populations.

Despite very promising results, the body of evidence from these 15 studies is still in its early stages, largely proof-of-concept and suffers from serious methodological limitations that prevent definitive clinical recommendations. Sample sizes were consistently small (median 108; only one study had more than 4000 exposures), follow-up rarely exceeded six months, and 14 of the 15 studies lacked randomization or a control group; therefore, the observed improvements cannot be causally attributed to the chatbots.

Only four studies reported a confirmed reduction in depression scores or obstetric complications, and most studies relied on proxy measures such as user satisfaction and message volume. Participants were predominantly urban, educated, and smartphone-owning women and rural, poorly educated, and migrant women the very groups that bear the greatest burden of maternal mortality were systematically excluded. Safety assessments were largely absent; No studies systematically documented critical errors, model confounding or false reassurance in high-risk settings. Geographic coverage was also heavily skewed toward upper-middle-income and high-income countries, and there were no trials from sub-Saharan Africa or South Asia regions that account for 94% of global maternal deaths. Consequently, despite exceptional user acceptance and engagement, the current evidence base does not yet support the use of this technology as a stand-alone intervention, and emphasizes the urgent need for large randomized trials with hard clinical outcomes, rigorous safety assessments and deliberate inclusion of more vulnerable populations before widespread implementation. None of the 15 included studies were randomized controlled trials, and all were at the feasibility, prototyping or user experience evaluation stage. This is the most important limitation of the current evidence and underscores the need for large, multicenter RCTs with hard clinical outcomes. Despite encouraging results in user acceptance and usability, current evidence is largely at the proof-of-concept stage and small single-arm studies, and still lacks large randomized trials, hard clinical outcomes, long-term follow-up and safety assessment in real high-risk populations. Therefore, this technology could be helpful as a powerful adjunct to human monitoring.

## Conclusion

LLM/GPTs and chatbots hold transformative potential for maternal and newborn healthcare, bridging critical gaps in traditional care through accessible information, emotional support and advanced diagnostic capabilities. Despite this, current limitations like readability challenges and ‘hallucinations’ mean they are not direct replacements for human medical professionals. The most promising path forward involves hybrid approaches, merging structured reliability with dynamic AI for personalized, adaptive and safe support. Crucial human oversight and transparent communication of these tools’ limitations are paramount for responsible integration. Adoption of WHO-aligned reporting standards and digital health registries will enhance replicability and global knowledge sharing. Ultimately, these powerful aids are designed to augment, not replace, human healthcare services, empowering expectant families and providers for a healthier future.

### Declaration

The authors confirm that the conception, design, data collection, analysis and interpretation of this work were conducted entirely by the authors and final edited by native language expert. Artificial intelligence tools (ChatGPT, GPT-4, OpenAI) were used solely to support language refinement, grammar correction and translation of the authors’ originally written content English. These tools were not used to generate original scientific ideas, data, analyses or conclusions.

## Data Availability

The datasets used and/or analyses during the current study available from the corresponding author on reasonable request.
